# Complete Genome Sequence of a Newcastle Disease Virus Isolated from Wild Peacock (*Pavo cristatus*) in India

**DOI:** 10.1128/genomeA.00495-14

**Published:** 2014-06-05

**Authors:** Sagar A. Khulape, Satish S. Gaikwad, Madhan Mohan Chellappa, Bishnu Prasad Mishra, Sohini Dey

**Affiliations:** a Division of Veterinary Biotechnology, Indian Veterinary Research Institute, Izatnagar, Uttar Pradesh, India; b OIE Reference Laboratory for Newcastle Disease, Gyeonggi-do, South Korea

## Abstract

We report here the complete genome sequence of a Newcastle disease virus (NDV) isolated from a wild peacock. Phylogenetic analysis showed that it belongs to genotype II, class II of NDV strains. This study helps to understand the ecology of NDV strains circulating in a wild avian host of this geographical region during the outbreak of 2012 in northwest India.

## GENOME ANNOUNCEMENT

Newcastle disease (ND) is a listed disease of the World Organisation for Animal Health affecting various avian species, with wide geographical distribution. The disease is caused by avian paramyxovirus serotype 1 (APMV-1) or Newcastle disease virus (NDV), the prototype of the *Avulavirus* genus of the *Paramyxoviridae* family. Wild birds have constantly been implicated in the spread of ND to domestic bird populations (1). In 2012, an NDV outbreak caused significant fatality in a wild peacock population in the northwest region of India (2). The natural habitats of peacocks are streamside and cultivated areas. The species is also known to forage around human habitats and villages for scraps. The movement of this wild species in close proximity to domestic birds, most of which are reared as backyard poultry, always threatens to the point of becoming a disease outbreak.

A necropsy sample comprised of brain tissue of a dead peacock suspected to have ND from the wild was received from Rewari of Haryana state, India. Virus identification was done by conventional diagnostic methods, such as virus isolation in 9-day-old specific-pathogen-free embryonated chicken egg hemagglutination test, which was further confirmed by a molecular diagnostic test, reverse transcription-PCR (RT-PCR).

Complete genome sequencing of the isolate was done using an overlapping RT-PCR strategy (3) to cover the full-length genome of the isolate (ABI 3730 DNA analyzer; Applied Biosystems). The sequence editing and assembly were done using the Serial Cloner program, and the isolate was referred to as NDV/Peacock/India/2012. The total length of the viral genome was found to be 15,186 nucleotides, similar to that of other NDV strains from early genotypes found during 1930 to 1960 (1). Genome sequencing revealed the presence of a polybasic amino acid motif ^111^GRRQKRF^117^ at the fusion protein cleavage site (FPCS), a primary molecular determinant of virulence for NDV, characteristic of virulent viruses (4). The vulnerability of wild peacocks in this case may be due to the absence of neutralizing antibodies, as the country lacks a dedicated wild avian vaccination program.

The phylogenetic relationship of NDV/Peacock/India/2012 with 54 previously characterized AMPV-1 strains from the Asian continent and other parts of the world was analyzed using the BEAST software package (5). The maximum clade credibility tree of the completed genome scale data placed the isolate in genotype II, class II of APMV type I viruses with the highest posterior probability value. The isolate was also placed as a different clade in genotype II viruses, as evidenced by the mean percent nucleotide sequence distance for the complete genome (5.7%), as determined by pairwise sequence comparison analysis (PASC) using the MEGA5 software (6).

The elucidation of the complete genome of the viral isolate will aid in the development of a vaccine candidate against homologous genotypes that are in circulation in India.

### Nucleotide sequence accession number.

The complete genome sequence of NDV/Peacock/India/2012 has been deposited in GenBank under the accession no. KJ769262.
